# Immersive Virtual Reality Applications in Schizophrenia Spectrum Therapy: A Systematic Review

**DOI:** 10.3390/ijerph17176111

**Published:** 2020-08-22

**Authors:** Emanuele Bisso, Maria Salvina Signorelli, Michele Milazzo, Marilena Maglia, Riccardo Polosa, Eugenio Aguglia, Pasquale Caponnetto

**Affiliations:** 1 Department of Clinical and Experimental Medicine, Psychiatry Unit, University Hospital “Policlinico G.Rodolico - San Marco”, University of Catania, 95123 Catania, Italy; eugenio.aguglia@unict.it; 2 Center for Tobacco Prevention and Treatment, University Hospital “Policlinico G.Rodolico - San Marco”, University of Catania, 95123 Catania, Italy; michele-milazzo@outlook.com (M.M.); m.maglia@unict.it (M.M.); polosa@unict.it (R.P.); 3 Department of Clinical and Experimental Medicine, Center of Excellence for the acceleration of Harm Reduction (COEHAR), University of Catania, 95123 Catania, Italy

**Keywords:** VR, immersive, virtual reality, schizophrenia spectrum, therapy, digital technologies, systematic review

## Abstract

(1) *Background*: Virtual Reality (VR) is a fully immersive computer simulated experience consisting of a three-dimensional interactive virtual environment, through a head-mounted display (HMD) and controller. The use of virtual reality has recently been proposed for the treatment of various psychiatric conditions, including the spectrum of schizophrenia. Our review aims to investigate the current available evidence regarding the use of immersive virtual reality in the treatment of psychotic symptoms. (2) *Methods*: From April 2019 to June 2020, we conducted a systematic review aimed at identifying therapeutic applications in immersive virtual reality for the spectrum of schizophrenia, searching for relevant studies on Web of Science, EMBASE, PsycINFO and CINHAL. (3) *Results*: We identified a total of 2601 unique records. Of these, 64 full-text articles were assessed for eligibility, and six out of these met the inclusion criteria and were included in the final systematic review. (4) *Conclusions*: The available data on immersive virtual reality are currently limited due to the few studies carried out on the topic; however, it has demonstrated its effectiveness and versatility in successfully treating various psychotic symptoms including delusions, hallucinations, or cognitive and social skills. Existing literature agrees on safe, tolerable, and long-term persistence of the therapeutic effects obtained by immersive VR. No serious side effects have been reported. In some specific cases, VR therapy was found to be very effective compared to usual treatment, allowing effective drug free interventions, and therefore without side effects for patients, even in those resistant to normal drug therapies.

## 1. Introduction

Virtual Reality (VR) is a fully immersive computer simulated experience consisting of the development of a three-dimensional virtual environment, around an individual through a head-mounted display (HMD). Such an environment is built to let the user look around him and move in every direction. It is also possible to interact with the environment through inputs given via a controller or keyboard or, in the most advanced VR headsets, through tactile gloves or body motion detection techniques. A subject’s motions are continuously detected so to adjust the 3D environment accordingly, to offer the individual the feeling of being immersed in a virtual space. VR technology has been around for a long time now. VR headset kits have been improved and miniaturized overtime. A few years ago, VR technology has become available on the consumer market at affordable prices. The modern VR kits are designed in order to be integrated with personal computers, gaming consoles or even smartphones.

VR has shown from the beginning a great potential and was successfully implemented in various fields, for instance, in industrial and professional design [1,2], but also in higher education [2] or training [3] According to the literature, possible fields of application of VR in medicine include patients’ rehabilitation [4,5,6] such as in case of Traumatic Brain Injury [7] or Parkinson’s disease [8], disability management [4], surgical training [4], psychological diseases therapy [4], and treatment of acute and chronic pain [4,5,6,7,8,9].

With specific regard to psychiatry and psychology, VR has already been applied to the treatment of various pathological conditions. Several clinical studies have demonstrated its efficacy in the treatment of post-traumatic stress disorder, anxiety disorder and specific phobias [10,11,12] such as flying phobia, height phobia and acrophobia [13]. Nevertheless, an immersive VR-based treatment of psychotic symptoms is a recently introduced research topic. In 2008, Freeman [14] first introduced the concept of VR applied to the treatment of psychosis but the first clinical trial was developed in 2016 by Freeman et al. [15]. VR has the ability to represent social environments that trigger responses, reactions and emotions equivalent to what the real world in a given context would create in patients’ mind [14,15,16,17], in similar way, a virtual person (avatar) will elicit reactions similar to those raised in real life [18,19]. This makes VR an ecologically valid environment for the assessment and treatment of psychotic disorders. Nowadays, relatively few studies have been conducted yet, nevertheless, according to the available literature, the wide range of potentially treatable target symptoms suggests the great potential and versatility of VR treatments in the field of schizophrenia spectrum disorders (SSD), meaning by this expression those included in the homonymous chapter “Schizophrenic spectrum disorders and other psychotic disorders” of the DSM-5.

Over the last years, some reviews were conducted on VR applications in schizophrenia and psychotic disorders [20,21,22,23] that suggested the validity of VR like treatment option. Veling et al. [20] stated that VR represents a good tool for assessment and treatment of psychotic disorders, although they believe that further studies are needed. Valmaggia et al. [21] underlined that some methodological limitations affect the studies about VR, such as small sample size, high dropout rates and lack of long-term follow-ups. According to their preliminary findings, Rus-Calafell et al. [22] suggested that VR can be applied to cognitive rehabilitation, social skills training interventions and virtual reality-assisted therapies for psychosis, offering a valuable method for assessing the presence of symptoms in ecologically valid environments, with the potential to facilitate learning new emotional and behavioral responses. Nevertheless Rus-Calafell [22] found only two articles on immersive VR in schizophrenia. Finally, Park et al. [23] recently reviewed (2019) the available evidence on VR applications in dementia, mild cognitive impairment (MCI), schizophrenia and autism, and found how VR therapies help patients to improve their quality of life.

It should be noted that two types of approaches can be recognized. The first one is “immersive” VR, commonly defined as VR, and the second one is non-immersive or “interactive” VR. These approaches are methodologically different, since the immersive one uses a head-mounted display (HMD) which allows complete immersion and interactivity of the patient in the virtual environment, while in the so-called interactive one, the patient is placed in front of an ordinary display reproducing a virtual environment. In this case, the individual interacts with the VR environment through a video game-like controller. The second approach lacks the immersive features that HMD based VR has. It is important to underline that, among the aforementioned reviews, only Valmaggia et al. [21] and Park et al. [23] draw an explicit distinction between immersive and non-immersive virtual reality.

To the best of our knowledge, no reviews were conducted specifically on immersive virtual reality applications related to the treatment of schizophrenia spectrum diseases. Our aim is to focus specifically on immersive VR therapies for SSD providing an updated view of the current spectrum of available therapeutic techniques and their effectiveness in clinical practice.

## 2. Materials and Methods

### 2.1. Search Strategy

The systematic review was fully conducted according to PRISMA guidelines 2009 for Systematic Reviews by PRISMA Group [24]. The protocol was registered on PROSPERO, an international database of prospectively registered systematic reviews in health and social care managed by the Centre for Reviews and Dissemination, University of York (Registration number CRD 42020154251).

From April 2019 until the date of submission of the article (June 2020), the reviewers BE and MM searched the databases Web of Science, EMBASE, PsycINFO and CINHAL for relevant studies using the following search terms string: (“virtual reality” OR vr) AND (psychos* OR schizophrenia OR hallucination* OR delusion* OR paranoia OR “paranoid ideation” OR “positive symptom*” OR “negative symptom*” OR voice* OR “severe mental illness*”). The systematic review was fully conducted according to PRISMA guidelines 2009 for Systematic Reviews [24]. The electronic searching was supplemented by hand-searching of reference lists of the included review articles to identify any additional source.

### 2.2. Eligibility Criteria

We included every article written in English language without temporal restrictions concerning publication date, meeting the following criteria:

(1) Participants: patients with schizophrenia spectrum disorders.
(2) Intervention: any kind of VR-based intervention performed through immersive virtual reality (VR), carried out with HMD (head-mounted display).
(3) Comparison: therapy as usual (TAU), not VR based, or other types of VR interventions.
(4) Outcomes: We considered the outcomes social skills and cognition, cognitive deficit, persecutory delusions and paranoia, and auditory verbal hallucinations (AVH).
(5) Study design: clinical trials.

### 2.3. Data Extraction

The reviewers EB and MM extracted data using a format which included: characteristics of studies and samples, type of intervention, target symptoms to be treated, frequency and duration of the interventions, outcomes and their assessments, follow-up if present.

### 2.4. Risk of Bias Assessment

The risk of bias for the included studies was assessed with Cochrane risk-of-bias tool for randomized trials, version 2 (RoB 2) by Sterne et al. [25].

## 3. Results

### 3.1. Characteristics of the Included Studies

**Figure 1:**
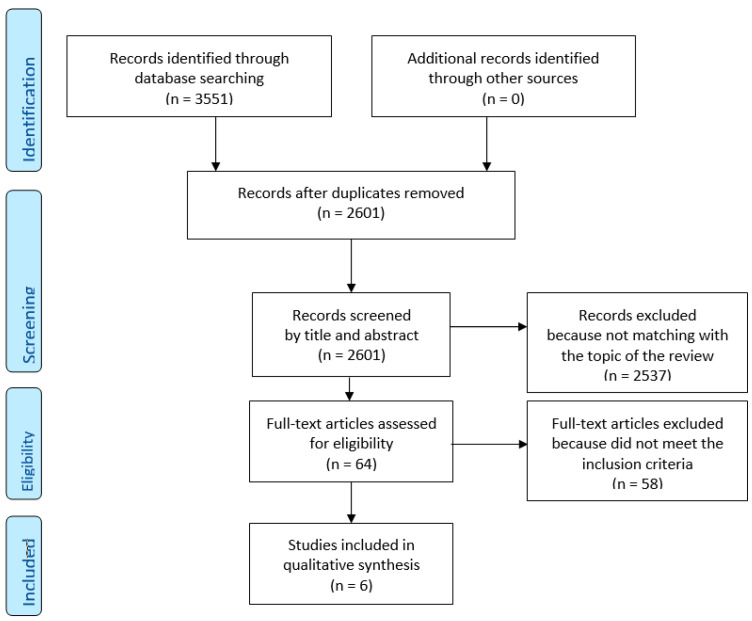
PRISMA (2009) Flow Diagram.

The database search identified a total of 3551 articles. After excluding duplicates, we found 2601 unique records, which were initially screened independently by reviewers BE and MM (please see section Author Contributions), based on title and abstract data. The screening has selected 64 articles to assess for eligibility criteria, 36 conflicts was resolved, 2537 records have been excluded because not matching with the intent of present review. Sixty-four full-text articles were assessed for eligibility, and six out of these met the inclusion criteria and were included in the systematic review, 58 were excluded because they did not deal with VR therapy or immersive VR (flow diagram, Figure 1). The included studies are summarized in Table 1, while in Table 2 we reported the various types of VR interventions. Risk of bias assessment for included studies is shown in Table 3. In La Paglia et al. (2016) the experimental and control group were not homogeneous in numerosity (*n* = 9 vs. *n* = 6) sex and age difference, as the control group is made up exclusively of males with an average age higher than the experimental group, these make the randomization process marked as high risk (Table 3).

#### 3.1.1. Delusions and Paranoia

We identified two randomized controlled trials on the treatment for delusions and paranoia: one based on VR exposure and VR (cognitive behavior therapy) CBT by Freeman et al. [15], the second one on VR CBT only, by Pot-Kolder et al. [26]. Freeman et al. starting from the idea that persecutory delusions derive from inconsistent threat beliefs that activate safety-seeking behaviors aimed to avoid anxiogenic situations [27], introducing virtual reality-controlled exposure as a possible therapy [14]. They developed these ideas in a subsequent trial that was included in this review [15]; in particular, they borrowed the controlled exposure already employed to treat phobias and anxiety, proposing for the first time the use of a VR version exposure therapy and CBT to treat delusions. In their pilot study, they compared the effect of VR exposure therapy to VR exposure modified with cognitive therapy elements across six VR scenarios, on a group of 30 patients affected by persecutory delusions, testing their effectiveness and confirming the initial idea about inconsistent threat beliefs. Their findings showed significative reduction in delusion’s conviction in both groups. VR CBT, however, has proved itself 22% more effective than VR exposure. They also demonstrated how these VR therapeutic results were still consistent after therapy in dealing with real-world situations, too. These results are further confirmed by Pot-Kolder et al. [26] that demonstrated how VR CBT can reduce significantly paranoid ideation in schizophrenic patients. Even in this case the results obtained with VR therapy were maintained in daily life and was recorded a decrease of safety behaviors and social cognitive problems specifically targeted by the authors. We have to conclude that the aforementioned trials support the effectiveness of VR CBT and VR exposure in the treatment of delusions and paranoia in schizophrenic patients, although in our opinion, it is necessary to expand the samples and make a trial confronting VR CBT with classic CBT.

#### 3.1.2. Auditory Verbal Hallucinations

We found a single RCT by du Sert et al. [28] regarding the treatment of auditory verbal hallucinations (AVH) through the VR Avatar therapy. The auditory verbal hallucinations represent one of the most frequent and disabling symptoms associated with psychosis. Avatar therapy was first introduced by Leff et al. [29] as non-pharmacological intervention method for AVH treatment. Leff et al. [29], however, did not make use of virtual reality but employed standard monitor visualization instead, so to administer “computer assisted therapy” without immersive characteristics. Avatar therapy with the implementation of immersive virtual reality, was successively introduced by du Sert et al. in 2018 [28] that, to our knowledge, is the only clinical trial conducted on treatment of AVH thought immersive VR to date. In this randomized partial cross-over trial, patients affected by pharmacologically resistant schizophrenia treated with VR showed significant improvements in AVH severity, depressive symptoms, and life quality with respect to those in the TAU control group. The sample consisted of 15 patients, including interventional and TAU groups. Patients’ improvements remained stable overtime and even got better at the 3-month follow-up, showing that the coping strategies learned during VR therapy applied to everyday life brought further improvements and enhanced life quality of these patients, even after the end of the therapy itself. The effectiveness of VR avatar therapy on AVH was supposed to be linked to the immersive VR environment capacity to induce feelings of presence, activating emotional arousal according to Diemer et al. [30], so that it is possible to work therapeutically on emotional dysregulation improving patients’ self-esteem and self-image. Emotional dysregulation was in fact recently identified as central principle in the treatment of psychotic symptoms by Trémeau [31], Khoury and Lecomte [32], and Craig et al. [33]. Through immersive VR, the patients can explore and experiment emotions in a safe controlled environment and learning how to better regulate them modifying their relationship with persecutory voices. These results, although preliminary, indicate how VR Avatar therapy could represent an innovative and useful non-pharmacological therapeutic intervention tool for hallucinations, especially in case of drug-resistant schizophrenia, which is otherwise poorly treatable.

#### 3.1.3. Cognitive Deficits

Two clinical trials by La Paglia et al. [34,35] were focused on treatment of cognitive deficits thought VR training. It consists in training the patient through targeted exercises performed in VR scenarios that reproduce everyday life situations (see Table 2). The cognitive functions in schizophrenic patients are often compromised, and this heavily impacts subject’s functioning, especially in relation to social activities and self-sufficiency, and more generally in carrying out targeted activities.

In their first clinical trial (2013), La Paglia et al. [34] performed VR trainings for cognitive rehabilitation in schizophrenic patients, targeting attention and executive functions. It was based on tasks execution in four VR environments including a park, a supermarket, a beach and a valley. It should be noted that the type of VR equipment used was not specified in this article, although it looks reasonable to assume immersive virtual reality was used. They confronted a VR-exposed group with a control group treated with IPT (integrated psychological therapy) only. After 10 sessions, similar improvements marked both groups out, although they were more significant in the VR one, which showed better reduction of number of errors and tasks execution time, combined with an increased rules’ compliance and sustained attention. These results were confirmed by a second trial in 2016 [35], in which the same experiment was carried out using three VR environments (park, supermarket, and beach). In addition to what was recorded during the first trial, patients treated with VR training showed better improvements in sustained attention, general cognitive functioning and planning than those of the control group treated with IPT only. In conclusion, current findings suggest the effectiveness of such therapy, but results look only slightly better in VR-treated groups than what was recorded in the control groups; therefore, in our opinion, a bigger generalization on larger population samples is needed in order to validate VR treatments.

#### 3.1.4. Social Skills

We identified a single randomized controlled trial on the use of immersive virtual reality in the treatment of social skills by Park et al. [36], in which the effectiveness of social skill training intervention in VR (VR SST, see Table 1) versus traditional SST (TR-SST) was compared on a sample of 91 schizophrenic patients diagnosed according to DSM 4 criteria. The VR SST is based on social training and role-play interventions in an immersive virtual environment with avatars reproducing social interactions, while TR-SST is based on interactions with actors and audio-video material. According to the findings of this paper, VR SST demonstrated greater improvements in the conversational skills and also induced greater assertiveness and motivation to treatment than TR-SST. On the other hand, VR SST was less effective in improving vocal and non-verbal skills than classical TR-SST. Interactions with avatars instead of with real people, as in the TR-SST, and difficulties of the therapist in being able to observe and correct patient’s non-verbal language while wearing the VR viewer are the reasons why such a result was obtained.

## 4. Discussions

It is remarkable that among authors there is no general agreement and uniqueness on the use of the term “virtual reality”, but often a general confusion, probably due to its recent introduction in medical research. Many authors in fact generically refer to “virtual reality” implying both the immersive and non-immersive types without clear distinctions, whereby “immersive” we mean the VR that is administered through head mounted display (HMD), characterized by total and natural immersion of the patient in the virtual environment, while by “non-immersive” we mean that is administered through a panoramic or classic screen and interactive commands so, unlike the first one, not to allow a real and total immersion. The latter should not be defined in our opinion as real “virtual reality” because it lacks the characteristic of total immersion that specifically differentiates this novel medium. The concept of non-immersive virtual reality gets too close to the experience of a classic video game played on a console or PC. In fact, such a concept has the defect of excluding immersivity. Immersive VR with adequate equipment allows a greater sense of reality in a virtual environment, since virtual scenes through HMD follow the head movements, while in non-immersive VR, it is possible to simply move the camera on a flat screen like in any video game. Immersivity helps to reproduce and elicit through VR the same emotions and reactions that the subject would experience in the real world. In this sense, as mentioned in the introduction, VR proved to be an ecologically valid environment, giving the possibility to develop new perspectives of assessment and therapy, as well as standardizable and repeatable environments and situations, difficult or impossible to be recreated in the real world. What, in fact, identifies specifically and represents the novelty of VR is the union of full immersivity and interactivity in a single instrument. By exploiting the union of the two, new scenarios and possibilities can be considered in the treatment of mental disorders and schizophrenia, exploiting a virtual malleable “alternative reality”. For all these reasons, in order to avoid vagueness in the literature, in our opinion the term “virtual reality” should exclusively imply “immersive virtual reality”.

Just the highly standardization and reproducibility of virtual environments (not possible during a classical face-to-face patient visit) offers the key for a possible use of VR in the assessment of mental disorders. For example, as underlined by Freeman [14], it is possible recording the physiological correlates of symptoms during the exposition to a particular environment or social situation reproduced in VR, such as participant’s movement, eye movements, signs of arousal like skin conductance, heart rate and blood pressure, etc., or also studying brain activation thought magnetic resonance imaging during a VR session. Moreover, some parameters can be directly measured during the session, mapping the interactions between controlled virtual social environments in relation to symptom domains, physiological responses and behavior, allowing a more personalized and contextual diagnostic assessment to be made [20]. In the same way it is potentially possible to identify any environmental triggers that can induce or worsen the psychopathological symptoms.

According to our findings, immersive VR has some obvious advantages compared to conventional treatments: first, it allows effective and brief interventions without the use of drugs and, therefore, the pharmacological side effects; second, it is highly customizable on the needs of the individual patient; third in some cases, it allows treatments of pharmacological resistance psychotic symptoms, too. We noticed that, in some case, the sample size is too small, and the trial was conducted with per-protocol approaches instead of intention-to-threat and, therefore, this may introduce bias. Looking at the future of immersive VR treatments, it is necessary to develop a greater degree of interactivity in order to make virtual environments and social interactions more and more complex, plausible and adherent to reality, at the same time these interactions must adapt and react to the behavior of the patient dynamically. In this sense, some protocols for future trials have already been published about forms of automated psychological VR therapy [37,38]. This involves automating the provision of cognitive therapy within VR using an avatar coach, so that a therapist is not needed [38]. New intervention protocols have also been proposed regarding social cognition and social functioning (DISCoVR) [39] and cognitive deficit [40]. Preliminary results were presented at the SIRS 2020 congress concerning the use of VR for the improvement of physical health [41] and social functioning [42] in schizophrenic patients. It is desirable to aim for greater diffusion of these technologies in hospital and outpatient settings thanks to the progressive decrease in costs, to meet the patient’s needs with increasingly scalable and personalized VR therapies. The future of VR treatments is currently promising and will face new challenges in the coming years. There is a general need to understand how new technologies, given their transformative potential, can find a place within the therapeutic practice [43].

## 5. Conclusions

In conclusion, the available data on immersive virtual reality are currently limited due to the few studies carried out on the topic and more research is needed to better evaluate their effectiveness as a treatment. However, the available evidence seems to suggest its effectiveness and versatility in successfully treating various psychotic symptoms including delusions, hallucinations, or cognitive and social skills, proposing itself as a promising new branch of research and therapy. The existing literature agrees on the safety, tolerability, and long-term persistence of the therapeutic effects obtained by immersive VR. No serious side effects have been reported, apart from temporary motion sickness or rare annoyance wearing VR equipment by Du Sert et al. [26].

Current evidence does not yet allow to establish whether VR treatments are better than conventional ones since, in some cases, results were similar or slightly better for VR. In some specific cases, VR therapy was found to be very effective compared to usual treatment, such in improving conversational skills. In VR-based treatment of AVH, results showed improvement at follow-up after the end of VR therapy as a result of the acquisition and real-life reinforcement of the behavioral patterns learned during the treatment. The fact that virtual reality is not necessarily better but has similar effectiveness, does not mean that it is not useful, since it has undoubted advantages that may make it the chosen therapy compared to conventional treatments, such as versatility, reproducibility, standardization of treatments, adaptability on patients’ needs, rapidity, effectiveness, long-term persistent results even after a few sessions, greater patient motivation and therefore it is characterized by an excellent cost-effectiveness ratio.

It should also be emphasized that immersive VR allows effective drug-free interventions and, therefore, without side effects for patients, even in those resistant to normal drug therapies, such as in the case of persistent AVH or negative symptoms. Today’s wide and growing availability of VR devices (like Oculus Rift or HTC Vive series) and their diffusion on the market make them potentially a powerful, cheap, and versatile tool of therapy for schizophrenia spectrum disorders. We must conclude that applications of immersive VR for treatment of psychotic disorders are very promising at present. However, in our opinion, due to the lack of studies on the argument, new ones on larger samples are needed to confirm the promising results obtained so far.

## Figures and Tables

**Table 1:** Articles included in the review (sorted alphabetically by first author).Legend: BAVQ-R = Beliefs About Voices Questionnaire-Revised; BDI = Beck Depression Inventory; BAI = Beck Ansiety Inventory; ESM = Experience Sampling Method; WCST (Winsconsin Card Sorting Test); FAB = Frontal Assessment Battery; MMSE = Mini-Mental State Examination; PANSS = Positive and Negative Syndrome Scale for schizophrenia; PSYRATS = Psychotic Symptom Rating Scales; QLESQ-SF = Quality of Life Enjoyment and Satisfaction Questionnaire-Short Form; SBQ-PB = Safety Behaviors Questionnaire-Persecutory Beliefs; SBS = Social Behavior Scales; TMT-A = Trial Making Test form A; TMT-B = Trial Making Test form B; TMT B-A = Trial Making Test form B-A; ToL = Tower of London test); WCST = Winsconsin Card Sorting Test.

Authors	Year	Title	Target	Country	Study Design	Total Sample	Patient’s Diagnosis	DSM	Type of VR-Therapy	Number of Sessions	Duration of Each Session	Duration of Treatment	Principal Outcome Assessment	Principal Findings
du Sert, O. P.; Potvin, S.; Lipp, O.; Dellazizzo, L.; Laurelli, M.; Breton, R.; Lalonde, P.; Phraxayavong, K.; O’Connor, K.; Pelletier, J.-F.; Boukhalfi, T.; Renaud, P.; Dumais, A.	2018	Virtual reality therapy for refractory auditory verbal hallucinations in schizophrenia: A pilot clinical trial	Auditory verbal hallucinations	Canada	randomised controlled trial	15	schizophrenia or schizoaffective disorder	DSM-5	AVATAR therapy	1 avatar creation session + 6 sessions	45 min	7 weeks	PSYRATS BAVQ-R PANSS QLESQ-SF	VRT produced significant improvements in auditory verbal hallucinations severity, depressive symptoms and quality of life that remained table at the 3-month follow-up period.
Freeman, D.; Bradley, J.; Antley, A.; Bourke, E.; DeWeever, N.; Evans, N.; Černis, E.; Sheaves, B.; Waite, F.; Dunn, G.; Slater, M.; Clark, D. M.	2016	Virtual reality in the treatment of persecutory delusions: Randomised controlled experimental study testing how to reduce delusional conviction	Delusions and paranoia	UK	randomized controlled trial	30	psycosis with persecutory delusions	not specified	VR-CBT and VR-exposure	1	30 min	1 day	PANSS PSYRATS BDI BAI SBQ-PB	Cognitive therapy using virtual reality could prove highly effective in treating delusions. In comparison with exposure, virtual reality cognitive therapy led to large reductions in delusional conviction (reduction 22.0%, and real-world distress (reduction 19.6%,).
La Paglia F.; La Cascia C.; Rizzo R.; Sanna M.; Cangialosi F.; Sideli L.; Francomano A.; Riva G.; La Barbera D.	2016	Virtual reality environments to rehabilitation attention deficits in schizophrenic patients	Cognitive deficits	Italy	clinical trial	15	schizophrenia	DSM-5	VR-training	10	90 min	10 weeks	MMSE FAB TMT-A, TMT-B, TMT B-A ToL Memory Battery WCST	Both VR training and IPT were associated with improved performance. VR training was additionally related with better cognitive functioning (MMSE), and with improved planning (TOL), and sustained attention (TMT-A).
La Paglia, F.; La Cascia, C.; Rizzo, R.; Sideli, L.; Francomano, A.; La Barbera, D.	2013	Cognitive Rehabilitation of Schizophrenia Through Neurovr Training.	Cognitive deficits	Italy	clinical trial	12	schizophrenia	DSM IV-TR	VR-training	10	90 min	10 weeks	MMSE ToL FAB TMT-B execution time, error number	VR may improve cognitive functioning in psycotic patients.
Park, K.-M.; Ku, J.; Choi, S.-H.; Jang, H.-J.; Park, J.-Y.; Kim, S. I.; Kim, J.-J.	2011	A virtual reality application in role-plays of social A randomized, controlled trial	Social skills	Korea	randomized controlled trial	91	schizophrenia	DSM-IV	SST-VR	10	90 min	5 weeks	SBS	SST-VR group improved more in conversational skills and assertiveness.
Pot-Kolder, Roos M. C. A.; Geraets, Chris N. W.; Veling, Wim; van Beilen, Marije; Staring, Anton B. P.; Gijsman, Harm J.; Delespaul, Philippe A. E. G.; van der Gaag, Mark;	2018	Virtual-reality-based cognitive behavioural therapy versus waiting list control for paranoid ideation and social avoidance in patients with psychotic disorders: A single-blind randomised controlled trial	Delusions and paranoia	Holland	randomised controlled trial	116	psychotic disorder and paranoid ideation	DSM-IV	VR-CBT	16	60 min	8-12 weeks	ESM (structured diary) via PsyMate electronic device	A large reduction was noted in momentary paranoia in the VR-CBT group, whereas a slight increase was noted in the control group; A significantly larger decrease in momentary anxiety was noted in the VR-CBT group than in the control These result remained significant at follow-up. Treatment effects on paranoid ideation were significant: at the post-treatment and follow-up assessments, levels of ideas of persecution and social reference were lower in the VR-CBT group than in the ontrol group. The VR-CBT group had improvements in self-stigmatisation and social functioning at follow-up whereas the control group did not.

**Table 2:** Classification of VR intervention types.

Type of VR Intervention	Description
VR training	Training consisting of a series of 3D-VR scenarios reproducing daily life and related challenges, to training and improve cognitive skills of schizophrenic subjects.
VR SST	Virtual reality social skill training (VR SST), consist of a VR version of classic SST intervention, thought conversation training and role-plays focused on interpersonal communication. It aimed at enhancing verbal, non-verbal skills and social cognition, detecting and correcting the errors committed by the patient on multiple communication levels.
VR Avatar therapy	The therapy involves a three-way conversation between therapist, patient and a VR digital simulation (avatar) of one of his hallucinated voices, made by patients himself as a representation of the entity to which he attributes his auditory hallucinations. The therapist’s voice is transformed through speech transformation software to coincide with the vocal characteristics attributed by the patient to the entity.
VR CBT (cognitive behavior therapy)	Use of techniques of cognitive behavioral psychotherapy (CBT) for the treatment of delusional disorders and paranoia, taking advantage of the immersive environment offered by VR, that allows a controlled exposure to social situation.
VR exposure	Therapy based on controlled exposure in a virtual reality environment to a situation or a stimulus that usually causes anxiety and fear in the subject, in order to induce a progressive desensitization towards it.

**Table 3:** Risk of bias assessment for included studies.

	Randomization process	Deviations from intended interventions	Missing outcome data	Measurement of the outcome	Selection of the reported result	Overall			
Du Sert et al. (2018)	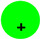	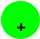	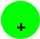	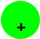	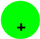	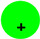			
Freeman et al. (2016)	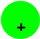	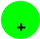	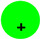	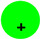	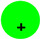	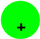			
La Paglia et al. (2013)	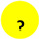	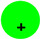	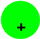	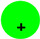	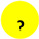	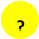			Legend:
La Paglia et al. (2016)	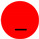	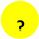	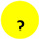	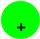	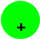	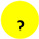		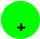	Low risk
Park et al. (2011)	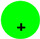	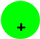	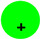	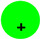	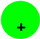	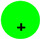		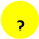	Some concerns
Pot-Kolder et al. (2018)	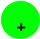	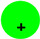	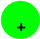	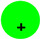	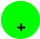	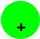		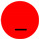	High risk
